# *LINC01605* Is a Novel Target of Mutant p53 in Breast and Ovarian Cancer Cell Lines

**DOI:** 10.3390/ijms241813736

**Published:** 2023-09-06

**Authors:** Michela Coan, Martina Toso, Laura Cesaratto, Ilenia Rigo, Silvia Borgna, Anna Dalla Pietà, Luigi Zandonà, Lorenzo Iuri, Antonella Zucchetto, Carla Piazza, Gustavo Baldassarre, Riccardo Spizzo, Milena Sabrina Nicoloso

**Affiliations:** 1 Division of Molecular Oncology, Department of Translational Research, Centro di Riferimento Oncologico di Aviano (CRO) IRCCS, Via Franco Gallini 2, 33081 Aviano, Italy; 2 Department of Mathematics, Informatics and Physics, University of Udine, Via delle Scienze 206, 33100 Udine, Italy; 3 Division of Clinical and Experimental Onco-Hematology, Department of Translational Research, Centro di Riferimento Oncologico di Aviano (CRO) IRCCS, Via Franco Gallini 2, 33081 Aviano, Italy

**Keywords:** mutant p53, lncRNAs, gain-of-function, breast cancer

## Abstract

*TP53* is the most frequently mutated gene in human cancers. Most *TP53* genomic alterations are missense mutations, which cause a loss of its tumour suppressor functions while providing mutant p53 (mut_p53) with oncogenic features (gain-of-function). Loss of p53 tumour suppressor functions alters the transcription of both protein-coding and non-protein-coding genes. Gain-of-function of mut_p53 triggers modification in gene expression as well; however, the impact of mut_p53 on the transcription of the non-protein-coding genes and whether these non-protein-coding genes affect oncogenic properties of cancer cell lines are not fully explored. In this study, we suggested that *LINC01605* (also known as *lincDUSP*) is a long non-coding RNA regulated by mut_p53 and proved that mut_p53 directly regulates *LINC01605* by binding to an enhancer region downstream of the *LINC01605* locus. We also showed that the loss or downregulation of *LINC01605* impairs cell migration in a breast cancer cell line. Eventually, by performing a combined analysis of RNA-seq data generated in *mut_TP53*-silenced and *LINC01605* knockout cells, we showed that *LINC01605* and mut_p53 share common gene pathways. Overall, our findings underline the importance of ncRNAs in the mut_p53 network in breast and ovarian cancer cell lines and in particular the importance of *LINC01605* in mut_p53 pro-migratory pathways.

## 1. Introduction

*TP53* is the most frequently mutated gene in human cancers, with breast and ovarian cancer exhibiting one of the highest mutation rates [1]. The *TP53* gene encodes for the tumour suppressor p53, a transcription factor that plays a critical role in several biological processes (e.g., cell cycle, apoptosis, senescence and DNA repair) [2,3,4,5,6]. Unlike other tumour suppressor genes, such as *BRCA1*, which are inactivated by truncating mutations or deletions, most *TP53* genomic alterations are missense mutations that lead to the production of a full-length protein with only one amino acid substitution [1]. Approximately 90% of these missense mutations occur at the DNA binding domain (DBD), impairing p53 stability and binding to its DNA responsive element within the promoters of transcriptional target genes, eventually resulting in the loss of wild-type p53 (wt_p53) tumour suppressor functions [7]. In addition to wt_p53 loss of functions, mutant p53 (mut_p53) enhances oncogenic features of cancer cells (gain-of-function activities) [8,9,10]: for instance, mut_p53 promotes the invasiveness of cancer cells by epithelial-mesenchymal transition (EMT) through the regulation of Zeb1 and Twist1 transcription factors [2,6,11,12].

The central dogma of molecular biology states that DNA is transcribed to messenger RNA, which in turn codes to proteins that have catalytic activities necessary for life. At the same time, despite the evident differences among metazoans (e.g., animals), the total number of protein-coding genes did not expand throughout evolution. Moreover, less than 2% of the human genome codes for proteins, while most of the human genome is transcribed, and non-protein-coding parts have been conserved during evolution. Former evidence suggests that the non-protein-coding part of the human genome may be a key factor in the regulation of biological processes. Transcribed non-protein-coding RNAs (ncRNAs) are grouped according to the length of their RNA molecules into small (about 25 nucleotides, such as microRNAs) or long non-coding RNAs (hundreds up to thousands of nucleotides). Long ncRNAs (lncRNAs) are non-coding transcripts longer than 500 base pairs (bp) [13], which can exert different cellular and biological functions including regulation of chromatin structure, gene expression, cell growth and differentiation. Deregulation of lncRNA expression has been associated with different tumours [14,15,16,17,18,19,20], and some lncRNAs have also been implicated in the wt_p53 regulatory pathway [21,22]. For instance, *LincRNA-p21* was the first lncRNA identified to be regulated by wt_p53, and it was reported to trigger apoptosis by acting as a transcriptional repressor [23]. On the other hand, increased expression of *MALAT1* in lung cancer was found to enhance cell proliferation and metastasis by downregulating wt_p53 targets [24].

To date, however, few studies have explored the relationship between mut_p53 gain-of-function and lncRNAs [25,26]. To identify novel lncRNAs regulated by mut_p53, we performed RNA sequencing in a *mut_TP53*-silenced breast cancer cell line. By this means, we identified *LINC01605* as a lncRNA regulated by mut_p53, and we investigated whether *LINC01605* participates in mut_p53 oncogenic functions using different functional assays and gene expression analysis.

## 2. Results

### 2.1. Mutant p53 Confers a Pro-Invasive Phenotype in MDA-MB-231 Cell Lines

**Figure 1:**
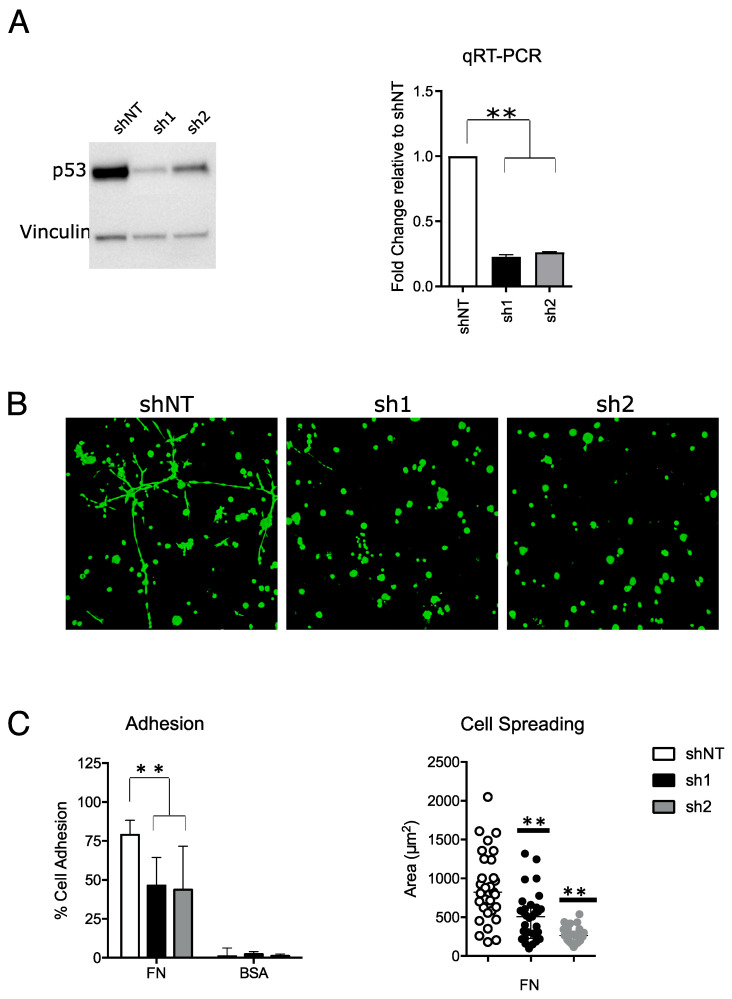
Mutant p53 confers pro-invasive phenotype in MDA-MB-231 breast cancer cell line. (**A**) Western blot (**left**) and qRT-PCR (**right**) of p53 in MDA-MB-231 shNT (shRNA not targeting), sh1 and sh2 at 9–10 days after shRNA transduction. Full-length blot is shown in Supplementary Figure S1A. (**B**) Representative 10× images of 3D Matrigel colony assay obtained with fluorescence confocal microscopy after calcein staining. Images were acquired 10 days following cell plating. (**C**) Left—histogram showing the percentage of cells that adhered to fibronectin (FN)-coated or control bovine serum albumin (BSA)-coated surface. Right—scatter plot showing the area of each single cell that adhered to FN-coated surface. Results are the mean of three independent biological replicates (** *p*-value ≤ 0.01).

Mut_p53 is known to promote tumour invasiveness and metastasis [2,3,4,6]. To recapitulate these effects in a cancer cell model, we selected a basal breast cancer cell line (i.e., MDA-MB-231) that carries the p53 mutation R280K, one of the most frequent mutations of *TP53* found in cancer patients. First, we silenced *mut_TP53* in the MDA-MB-231 cell line by lentiviral transduction of two shRNAs (sh1 and sh2). Mut_p53 protein and RNA levels decreased by approximately 80% in both sh1 and sh2 compared with control cells (shNT) (Figure 1A and Supplementary Figure S1A). About four days after viral transduction, *mut_TP53*-silenced cells decreased their proliferation rate until 9–10 days later when cells started to grow again (Supplmentary Figure S1B). For this reason, we evaluated the impact of *mut_TP53* silencing on breast cancer cell invasiveness at 9–10 days after shRNA transduction in order to avoid possible confounding results due to impaired proliferation. We performed 3D colony formation assay in a Matrigel matrix, and we did not observe any differences in the number of colonies between control (shNT) and *mut_TP53*-silenced cells (Figure 1B). At the same time, MDA-MB-231 shNT cells formed star-like shape colonies that invaded the extra-cellular matrix, whereas *mut_TP53*-silenced cells formed round-shaped colonies, indicating a less invasive phenotype (Figure 1B and Supplementary Figure S1B). To further evaluate the metastatic ability of mut_p53 in MDA-MB-231 cells, we performed cell adhesion assay and observed that *mut_TP53*-silenced cells had a decreased ability to adhere and spread on fibronectin compared to control cells (Figure 1C). Together, these data confirmed that mut_p53 confers a pro-invasive phenotype to the MDA-MB-231 cell line by increasing its invasive and adhesion capabilities.

### 2.2. RP11-527N22.2 and LINC01605 Represent a Unique lncRNA Transcript Regulated by mut_p53 in Breast and Ovarian Cancer Cell Lines

**Figure 2:**
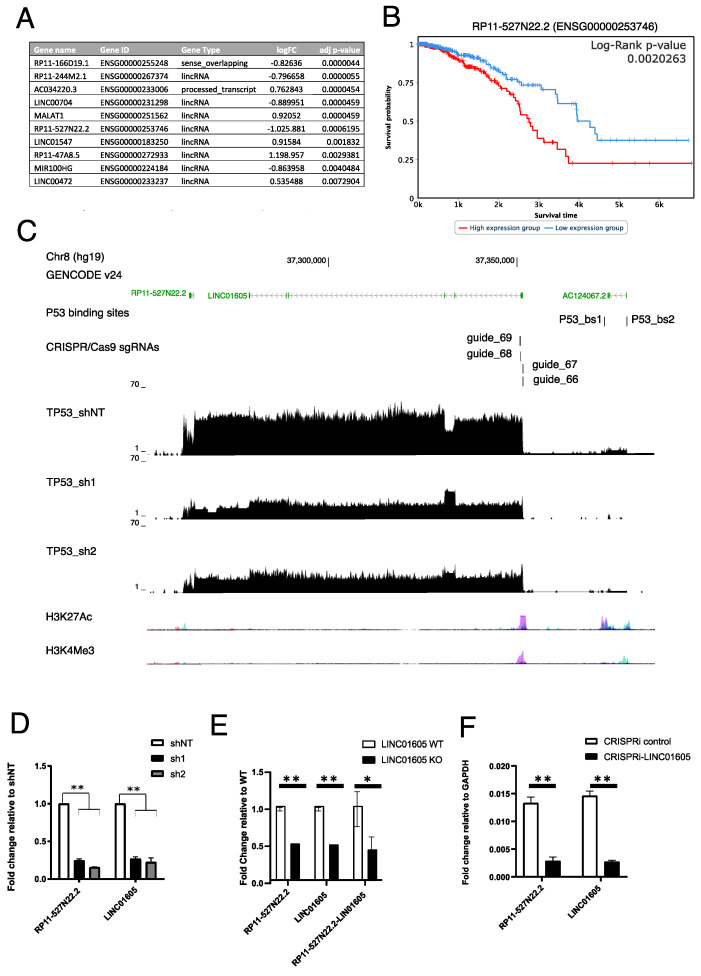
*RP11-527N22.2* and *LINC01605* lncRNAs represent a unique transcript. (**A**) Table showing top 10 lncRNAs differentially expressed in MDA-MB-231 sh1 and sh2 compared to shNT with their relative log2 fold-change (logFC) and adjusted *p*-value. (**B**) Kaplan–Meier plot showing overall survival of 942 patients carrying high or low *RP11-527N22.2* expressing tumours in the Breast Cancer—The Cancer Genome Atlas (BRCA-TCGA) dataset. (**C**) UCSC Genome Browser image showing the following from top to bottom: candidate mut_p53 binding sites near *LINC01605* locus (p53_bs1 and p53_bs2), CRISPR/Cas9 single guide RNAs (sgRNAs) targeting *LINC01605* first exon. BigWig files of RNA-seq reads from MDA-MB-231 shNT, sh1 and sh2 and ENCODE H3K27Ac and H3K4me3 histone mark tracks. (**D**) qRT-PCR showing *LINC01605* and *RP11-527N22.2* expression in shNT, sh1 and sh2 MDA-MB-231 cells. (**E**) qRT-PCR measuring expression levels of *LINC01605* and *RP11-527N22.2* and of a region spanning *LINC01605* and *RP11-527N22.2* in MDA-MB-231 cell WT and KO for the first exon of *LINC01605*. (**F**) RT-qPCR measuring expression levels of *LINC01605* and *RP11-527N22.2* in MDA-MB-231 cells transduced with *LINC01605-*CRISPRi (* *p*-value ≤ 0.05, ** *p*-value ≤ 0.01).

Evidence accumulated over the past years showed that lncRNAs can be regulated by wt_p53, and their deregulation might affect tumourigenesis and cancer dissemination [27]. For this reason, we investigated whether gain-of-function activities of mut_p53 could also regulate specific lncRNAs. Given that most lncRNAs are transcribed and retained in the nucleus where they can function as DNA regulatory elements, we measured lncRNA expression by RNA-seq in the nuclear fraction of control and *mut_TP53*-silenced MDA-MB-231 cells at 9–10 days after shRNA transduction. We found 1890 differentially expressed genes upon *mut_TP53* silencing by both sh1 and sh2 (log2 fold-change > |0.5|, padj < 0.05); of these, 1616 were annotated as protein-coding genes and 204 as ncRNAs. Among the ncRNAs, 99 were annotated as lncRNAs, out of which we selected the 10 with the lowest adjusted *p*-value (Figure 2A). Using real-time semi-quantitative PCR (qRT-PCR) on independent samples of *mut_TP53*-silenced MDA-MB-231 cells, we were able to validate 8 out of 10 candidate lncRNAs (Supplementary Figure S2A).

Among these, *RP11-527N22.2* was the most consistently downregulated (Supplementary Figure S2A), and it was the only candidate whose expression was associated with overall survival in breast cancer patients of The Cancer Genome Atlas (TCGA) dataset (Figure 2B), confirming previous results from Wei W. et al. [28]. For these reasons, we decided to further explore this transcript. To further validate that changes in *mut_TP53* expression levels impact the expression levels of *RP11-527N22.2*, we silenced *mut_TP53* in the OVCAR8 cell line, and we overexpressed R275H *mut_TP53* in the SKOV3 *TP53null* cell line [29]. In both models, we confirmed that *mut_TP53* regulates *RP11-527N22.2* expression (Supplementary Figure S2B,C).

By visualising the RNA-seq read mapping to the *RP11-527N22.2* locus in MDA-MB-231 shNT, sh1 and sh2, we noticed that transcription was a continuum between *RP11-527N22.2* and *LINC01605* (Figure 2C). This suggested that these two lncRNAs, which are annotated by GENCODE as separate genes, may represent a unique gene. In agreement with this hypothesis, the expression of both lncRNAs similarly decreased upon mut_*TP53* silencing (Figure 2C,D and Supplementary Figure S2C). To demonstrate that *LINC01605* and *RP11-527N22.2* are part of the same transcript, we generated a CRISPR/Cas9 knockout (*LINC01605*-KO) and a CRISPR/dCas9 KRAB (*LINC01605*-CRISPRi) model targeting the *LINC01605* first exon in MDA-MB-231 cells (Supplementary Figure S3A,B), and we investigated whether *LINC01605* and *RP11-527N22.2* RNA expression levels would concordantly decrease. As shown in Figure 2E,F, both *LINC01605*-KO and *LINC01605*-CRISPRi MDA-MB-231 cells displayed decreased expression of *LINC01605* and *RP11-527N22.2* and of a PCR amplicon linking the two lncRNAs (i.e., *RP11-527N22.2*-*LINC01605*) compared with parental cells (Figure 2E,F and Supplementary Figure S3C), demonstrating that the *LINC01605* first exon contains the transcription start site of a unique transcript that extends from *LINC01605* to *RP11-527N22.2*. Given the evidence that *RP11-527N22.2* and *LINC01605* represent the same transcript in MDA-MB-231 cells, from now on, we will name it *LINC01605*.

### 2.3. Identification of a Putative mut_p53-Dependent DNA Regulatory Element near LINC01605 Transcription Start Site

**Figure 3:**
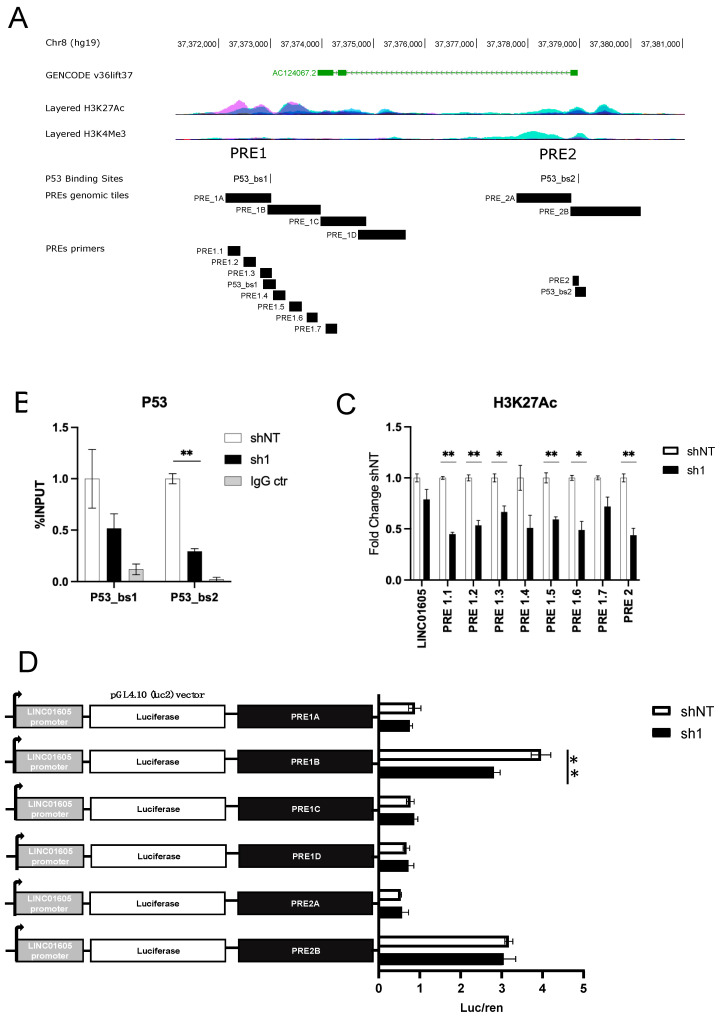
Identification of PRE1B as mut_p53-dependent enhancer, which regulates *LINC01605* expression. (**A**) UCSC Genome browser session displaying the following from top to bottom: PRE1 and PRE2 overlapping ENCODE H3K27Ac histone mark with putative binding sites for p53 (p53_bs1 and p53_bs2); genomic tiles for PRE1 (PRE1A, PRE1B, PRE1C, PRE1D) and PRE2 (PRE2A and PRE2B) with PRE primers for ChIP and RT-qPCR. (**B**) ChIP experiment by immunoprecipitating mut_p53 in MDA-MD-231 shNT and sh1 cells and qPCR enrichment at the newly identified mut_p53 binding sites (p53_bs1 and p53_bs2). (**C**) ChIP H3K27Ac enrichment in MDA-MB-231 shNT and sh1 cells at the *LINC016015* locus and at PRE1 and PRE2. Results are the mean of four technical replicates. (**D**) Luciferase reporter assay results in MDA-MB-231 shNT and sh1 cells for the PREs. Luciferase/renilla values were normalised to the pGl4.10-*LINC01605* promoter only construct. Results are the mean of three biological replicates. An empty pGL4.10 (luc2) vector was also transfected into cells to normalize luminescence values (not shown in the figure) (* *p*-value ≤ 0.05, ** *p*-value ≤ 0.01).

Following the identification of *LINC01605* as a candidate lncRNA regulated by mut_p53 in cancer cells, we investigated whether mut_p53 directly regulates *LINC01605* expression by looking for mut_p53 binding sites near the *LINC01605* locus. For this purpose, we re-analysed publicly available ChIP-seq data of mut_p53 in MDA-MB-231 cells [30]. We identified two putative mut_p53 binding sites (p53_bs) that are located 20 Kb downstream of the *LINC01605* first exon (p53_bs1 at chr8:37,372,992 and p53_bs2 at chr8:37,378,979, according to hg19 annotation). From now on, we refer to these candidate mut_p53 binding sites as putative regulatory elements (PREs)—PRE1 and PRE2—harbouring p53_bs1 and p53_bs2, respectively. According to ENCODE data, PRE1 and PRE2 overlap genomic regions enriched for the acetylation of lysine 27 of histone 3 protein (H3K27Ac), which is typically a hallmark of active DNA regulatory elements (Figure 3A). Consistent with these in silico findings, by performing ChIP-qPCR experiments in MDA-MB-231 cells, we confirmed mut_p53 binding and H3K27Ac enrichment, which both diminished upon *mut_TP53* silencing (Figure 3B,C).

To examine the potential transcriptional enhancing activity of PRE1 and PRE2 on the *LINC01605* promoter, we fragmented PRE 1 and 2 into different genomic tiles (tiles 1A, 1B, 1C and 1D for PRE1; tiles 2A and 2B for PRE2) (Figure 3A) that were sub-cloned into the pGL4.10 (luc2) vector containing the *LINC01605* core promoter sequence. We then performed luciferase reporter assays in shNT and sh1 MDA-MB-231 cells. Results showed that the genomic tiles 1B and 2B increased the transcriptional activity of the *LINC01605* promoter in vitro (Figure 3D); however, upon *mut_TP53* silencing by sh1, only the enhancing activity of 1B decreased, indicating that the transcriptional effect of the genomic tile 1B is mut_p53-dependent. In contrast, the enhancing activity of 2B was not influenced by mut_*TP53* downregulation even though it contained a mut_p53 binding site (mut_p53_bs2) (Figure 3D), suggesting that the binding of mut_p53 to p53_bs1 is sufficient to regulate *LINC01605* expression.

### 2.4. LINC01605 Regulates Breast Cancer Cell Migration

Having demonstrated that mut_p53 directly regulates *LINC01605* expression by binding to PRE1, we investigated the effect of *LINC01605* on oncogenic properties. To do so, we took advantage of MDA-MB-231 *LINC01605*-KO and *LINC01605*-CRISPRi cells that we previously described (Figure 2E,F and Supplementary Figure S3A,B). First, we tested whether *LINC01605* had an effect on cell proliferation: results showed that the KO of *LINC01605* very marginally inhibited population doubling in MDA-MB-231 cells (Supplemental Figure S4A).

**Figure 4:**
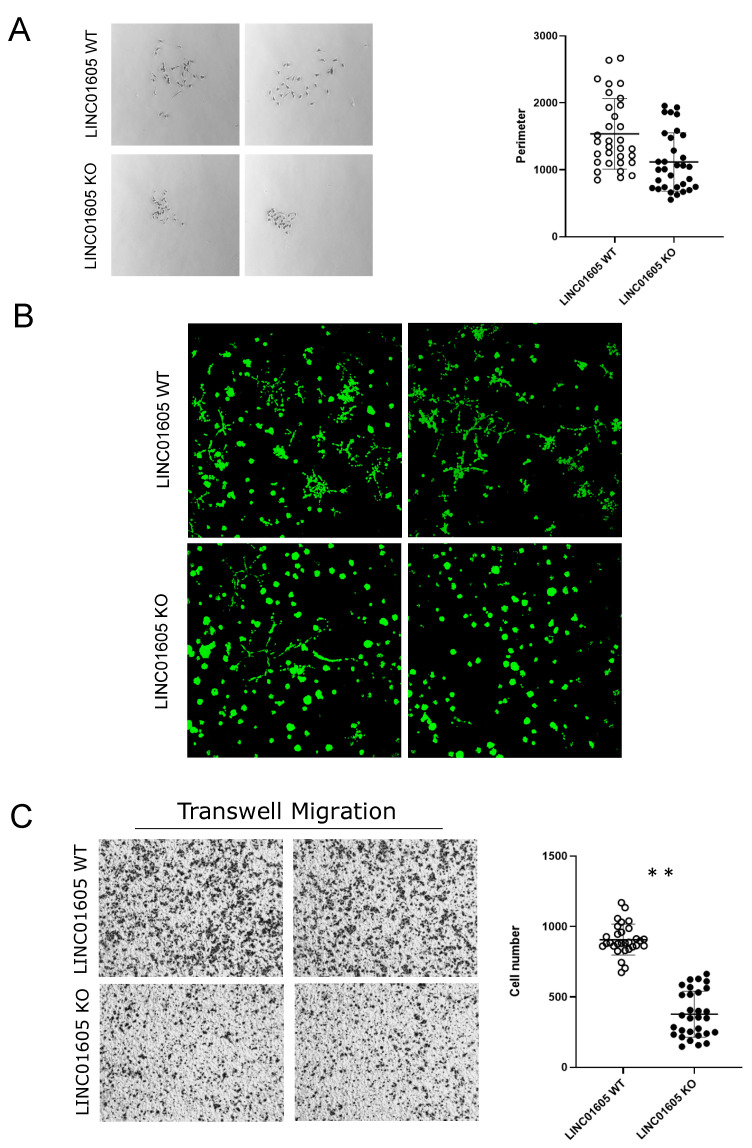
*LINC01605* oncogenic functions. (**A**) Left—representative 1× images of colonies formed by *LINC01605*-KO and *LINC01605*-WT cells Images were acquired with the stereomicroscope. Right—scatter plots showing the perimeter length of colonies formed by *LINC01605*-WT and KO clones using ImageJ. (**B**) Representative 10× images of 3D Matrigel colony assay obtained with fluorescence confocal microscopy after calcein staining. Images were acquired 10 days following cell plating. (**C**) Left—representative 1× images of cells migrated through fibronectin-coated Boyden chamber using the stereomicroscope. Right—count of migrated cells through Boyden chamber in MDA-MB-231 *LINC01605*-WT and KO cells. Images were acquired with the stereomicroscope. Fifteen different fields were acquired for each cell line and for each biological replicate (three independent biological replicates) (** *p*-value ≤ 0.01).

When we assesed the clonogenic ability of *LINC01605*-KO cells, we observed that *LINC01605*-KO cells formed tighter (less spread-out) colonies than their WT counterpart (Figure 4A), possibly indicating that *LINC01605* may regulate the motility of MDA-MB-231 cells. Thus, to investigate this *LINC01605* cell function, we explored the adhesion capability of *LINC01605*-KO cells on a fibronectin substrate without observing any significant difference (Supplementary Figure S4B). On the contrary, in a 3D colony formation assay in a Matrigel matrix, *LINC01605*-KO cells formed round-shape colonies compared with *LINC01605*-WT cells, which formed star-shape colonies, indicating that *LINC01605* increases the invasive ability of MDA-MB-231 cells (Figure 4B). To confirm the impact of *LINC01605* in cancer cell motility, we tested *LINC01605*-WT and KO cell ability to migrate across a transwell chamber using a fibronectin coating as an attractant. As shown in Figure 4C, *LINC01605*-KO cells migrated less than parental cells, which also happened in MDA-MB-231 cells upon *LINC01605*-CRISPRi transcriptional downregulation (Supplementary Figure S4C).

### 2.5. Pathway Analysis Reveals Resemblance between the Effect of mut_TP53 and LINC01605 in MDA-MB-231

To start exploring *LINC01605* function in cancer, we performed RNA-seq gene expression analysis in MDA-MB-231 *LINC01605*-WT and KO clones. We identified 532 differentially expressed genes upon *LINC01605*-KO (fold-change > |1.5| and *p*-value > 0.05), and to identify over-represented pathways, we used Gene Set Enrichment Analysis (GSEA) Pathway analysis [31,32]. Pathway analysis revealed EMT, apical junctions and myogenesis among the pathways enriched in *LINC01605*-KO cells compared with *LINC01605*-WT cells (Table 1). These pathways may explain the impact of *LINC01605* on invasion and migration that we observed in MDA-MB-231 cells (Figure 4A–C). When we compared enriched pathways in the RNA-seq profiling of *LINC01605*-KO cells with *mut_TP53*-silenced cells, we discovered that 13 out of the 16 (81%) pathways enriched in *LINC01605*-KO cells were in common with the pathways enriched in *mut_TP53*-silenced cells (Table 1).

In conclusion, herein, we described that *LINC01605* is an lncRNA directly regulated by mut_p53 through the binding to an enhancer region downstream of the *LINC01605* locus. We also showed *LINC01605′s* role in cell migration and resemblance with mut_p53 function. Eventually, by performing a combined analysis of RNA-seq data generated in *mut_TP53*-silenced and *LINC01605*-KO cells, we showed that *LINC01605* and mut_p53 share common gene pathways.

## 3. Discussion

In this study, we identified *LINC01605* as a novel lncRNA that is regulated by mut_p53 and that resembles several mut_p53 oncogenic properties (gain-of-function phenotype) in cancer cells. *LINC01605* was found to be downregulated upon mut_*TP53* silencing in MDA-MB-231 breast cancer and OVCAR8 ovarian cancer cells, and we confirmed that mut_p53 directly regulates *LINC01605* expression by binding to an enhancer region located 20 kb upstream of the *LINC01605* first exon. Furthermore, the loss of expression of *LINC01605* resulted in a marked reduction in the cell adhesion and migration capability of MDA-MB-231 cells, which recapitulates the phenotype of *mut_TP53* silencing in the same cell model.

So far, three studies have been published on *LINC01605* (which is also referred to as *lincDUSP*) demonstrating its oncogenic role in colon cancer and in laryngeal squamous cell carcinoma by promoting cell proliferation, migration and invasion [33,34,35]. These findings are consistent with the *LINC01605* pro-tumourigenic phenotype observed in our breast cancer model with some differences. For example, we only observed a slight inhibition of cell proliferation in MDA-MB-231 cells KO for *LINC01605*, and cell cycle analysis did not show any significant changes in cell cycle distribution in MDA-MB-231 cells with and without *LINC01605* expression. In addition, we did not observe differences in the clonogenic capacity of cells WT and KO for *LINC01605*, but rather *LINC01605-KO* cells exhibited a decreased invasion and migration capability in our migration experiments. These results, together with *LINC01605* capacity to regulate cell adhesion in MDA-MB-231 cells points to a possible *LINC01605* function in the EMT process, a key mut_p53 oncogenic feature [10,12,36]. Our in vitro findings could be also used as preliminary findings to plan in vivo experiments to further strengthen the translational aspects of our discovery.

Our hypothesis is further supported by the gene expression analysis of *LINC01605*-WT and KO cells, which revealed an over-representation of pathways linked to mut_p53 activities, including EMT and cell movement regulation. Additional studies are necessary to characterise other key downstream genes involved in this mut_p53-*LINC01605* regulatory axis.

Another feature that emerged from the integrative analysis of RNA-seq experiments from *mut_TP53*-silenced and *LINC01605-KO* MDA-MB-231 cells is that *LINC01605* seems to be involved in the dysregulation of lipid metabolism, similar to mut_p53 [37,38]. It is intriguing to speculate the existence of a novel relationship between mut_p53-dependent *LINC01605* oncogenic function and lipid metabolism that may be responsible for cancer progression. However, this hypothesis needs to be tested and further characterised.

In this work, we also found that *LINC01605* and *RP11-527N22.2* share the same promoter and are likely a unique transcript. This result is in contrast with GENCODE annotations in which the two lncRNAs are annotated as separate transcripts. For this reason, it would be interesting to extend *LINC01605* locus characterisation in different cancer cell models. This would allow us to gain a better knowledge of the *LINC01605* transcript and its regulation in different cancer types in which it acts as a potential oncogene.

So far, very few studies have investigated whether *mut_TP53* regulates and exerts its function via an lncRNA [25,26] and whether there is any clinical correlation. Altogether, our work gives new insights about the role of lncRNAs in the mechanisms underlying mut_p53 gain-of-function activities. In particular, our data suggest that *LINC01605* is a novel lncRNA directly regulated by mut_p53, involved in mut_p53-dependent increased cell invasion and motility in MDA-MB-231 cells and likely involved in cancer cell aggressiveness, since breast cancer patients with higher *LINC01605* expression levels display a worse outcome.

## 4. Materials and Methods

### 4.1. Cell Lines, Cell Cultures and Lentiviral Transduction

Cell lines and cell culture media used in this work are listed in Supplementary Table S1. Cell lines were maintained at 37 °C and 5% CO_2_ in humidified incubators.

*TP53*-silenced, KO *LINC01605* and *LINC01605-*CRISPRi MDA-MB-231 cells were all generated by lentiviral transduction [39]. Briefly, 293FT cells were co-transfected with the specific lentiviral vectors indicated in Supplementary Table S1 and two lentiviral packaging vectors (psPAX2 and VSV-G). Viral supernatants were collected 24, 36, 48 and 60 h after transfection and used to transduce MDA-MB-231 cells [39].

### 4.2. CRISPR-Cas9 System

*LINC01605-KO* and *LINC01605*-CRISPRi were generated using CRISPR/Cas9 system as described in [39]. To generate cell lines KO for *LINC01605*, four different single guide RNAs (sgRNA) (guides 66, 67, 68 and 69) (Supplementary Table S1) were used spanning approximately 800 bp of *LINC01605* first exon. Briefly, sgRNAs were cloned into pLV hUbC-Cas9-T2A-GFP. MDA-MB-231 cells transduced with this lentiviral system were sorted by flow cytometry in GFP− (WT) and GFP+ (KO) cells. To test CRISPR/Cas9 cut efficiency, genomic DNA was extracted using Gentra Puregene Cell Kit (Qiagen Sciences, Germantown, MD, USA) according to manufacturer’s instructions and sequenced using MiSeq system by Illumina (Illumina, San Diego, CA, USA). Sequencing reads were visualised using the Integrative Genomics viewer (IGV, Broad Institute, Cambridge, MA, USA) to confirm genetic deletion. Cell clones were grown after cell sorting by flow cytometry. DNA was extracted and clones screened by PCR, using specific primers (Supplementary Table S1).

### 4.3. ChIP Assay

ChIP assays to evaluate p53 and histone mark enrichment were performed using the SimpleChIP^®^ Enzymatic Chromatin IP Kit (Magnetic Beads) (Cell Signaling Technology, Inc., Danvers, MA, USA) following manufacturer’s protocol. The following ChIP-grade antibodies were used: anti-histone H3-acetyl K27, H3 tri-methyl K4, H3 methyl K4, H3 tri-methyl K9 (cat. No. ab4729, ab8580, ab176842, ab176916; Abcam, Cambridge, UK) and anti-p53 antibody DO-1 (sc-126 Santa Cruz Biotechnology, Inc., Santa Cruz, CA, USA). DNA levels for the regions of interest were quantified by qPCR using specific primers (Supplementary Table S1). Details regarding the number of technical and biological replicates are listed in the figure legends.

### 4.4. RNA Extraction, cDNA Synthesis and qRT-PCR

RNA was extracted using the Rneasy Plus Mini Kit (Qiagen Sciences, Germantown, MD, USA) according to protocol instructions. Extraction was followed by DNase digestion (Turbo-DNase, Ambion, Thermo Fisher Scientific, Waltham, MA, USA), and RNA quality was assessed by using agarose gel electrophoresis after RNA exposure to 70 °C for 5 min. cDNA synthesis was performed from 1 µg of RNA using the AMV Reverse Transcriptase with random primers (Promega, Madison, WI, USA). cDNA was then used for qRT-PCR reactions using iQ SYBR Green Supermix (Bio-Rad, Hercules, CA, USA) using the specific primers (Supplementary Table S1). qRT-PCR reactions were carried out in Micro seal^®^ 384-well PCR plates using the CFX384 Touch Real-Time PCR Detection system (Bio-Rad, Hercules, CA, USA). The 2^−∆∆Ct^ method was used to calculate the relative abundances of genes and regions of interest, measuring GAPDH expression as housekeeping control. Details regarding the number of technical and biological replicates are listed in the figure legends.

### 4.5. Luciferase Reporter Assay

*LINC01605* promoter (740 bp) was amplified from MDA-MB-231 genomic DNA using Phusion™ High-Fidelity DNA polymerase (Thermo Scientific, Waltham, MA, USA) and cloned into pGL4.10 [luc2] vector (Promega, Madison, WI, USA) in the multiple-cloning site (KpnI). MDA-MB-231 cells were transfected with this construct to test its activity compared to pGL4.10 [luc2] empty vector. PRE1 and PRE2 genomic tiles (1A, 1B, 1C and 1D for PRE1 and tiles 2A and 2B for PRE2) were cloned at the SalI restriction site of the pGL4.10 *LINC01605* promoter. All constructs were sequenced by Sanger sequencing to confirm their identity.

A reverse approach was used to transfect cells for luciferase reporter assays. Equimolar amounts of luciferase reporter plasmids, 50 ng of pRLTK plasmid (encoding renilla) and 1 µL of Lipofectamine 2000 (Invitrogen, Waltham, MA, USA) were mixed, and 100 µL of transfection mix was transferred into a 24-well plate. On top of the transfection mix, 2.5 × 10^5^ MDA-MB-231 cells were seeded per well in triplicate and incubated at 37 °C. The empty vector pUC19 was used to make the final amount of DNA (600 ng) constant between wells. Twenty-four hours after transfection, the luciferase/renilla activity was measured using the Dual Luciferase system (Promega, Madison, WI, USA) according to manufacturer’s instructions. The final values were obtained by dividing the luciferase values by the corresponding renilla values to control for variations in transfection efficiency. Luciferase/renilla ratios of all constructs were compared with pGL4.10 *LINC01605* promoter, and a two-way ANOVA test with Bonferroni’s correction for multiple comparisons was used to analyse the data using GraphPad Prism (Version 9.1.1, GraphPad, Inc., San Diego, CA, USA). Details regarding the number of technical and biological replicates are listed in the figure legends.

### 4.6. Gene Expression and ChIP-Seq Analysis

RNA sequencing was performed at IGA Technology Services Srl (Udine, Italy). Sequencing reads were aligned to GRCh37 reference assembly using HiSAT2. FeatureCounts was then used to count and assign reads to the genomic features in the GENCODEv24 annotation. Differential gene expression was performed using DESeq2 (fold-change > |0.5| and *p*-value > 0.05).

For p53 ChIP-seq analysis, we downloaded MDA-MB-231 IgG, Input and DO-1 (p53) fastq files from NCBI SRA database (accession number: SRX899076). Reads were aligned to GRCh37 human genome reference using BWA aligner 0.7.17. BAM files were then converted into bigwig files for data visualisation in UCSC Genome Browser. MACS2 in Galaxy (galaxyproject.org accessed on 9 February 2021) was used to estimate mut_p53 binding sites by setting the default minimum enrichment ratio value between 2 and 5, as the distribution of enrichment ratio of DO-1 versus Input had particularly low values.

### 4.7. Adhesion Assay, Colony Assay, Matrigel and Motility Experiments

For adhesion assays, 0.5 × 10^6^ cells were plated in 12-well plate wells coated with fibronectin (5 µg/mL) or BSA (1%) and incubated for 45 min at 37 °C (controls were fixed in 4% PFA immediately after plating). Following incubation, cells were washed with PBS 1× and fixed with 4% PFA. Cells were then stained with Crystal Violet, and images of wells were acquired using a microscope. The number of adherent cells was estimated by analysing images with ImageJ. For colony assay experiment, 1000 cells were plated in 10 cm cell culture dishes in triplicate and let to grow in complete medium at 37 °C. After one week, cell colonies were fixed with 4% PFA and stained with Crystal Violet. Cell colonies and appearances were counted by acquiring images with stereo microscope and analysing them using ImageJ (version 2.0.0, National Institutes of Health, Bethesda, MD, USA).

For the Matrigel experiment, 2000 cells were plated with 150 µL of Matrigel matrix (Geltrex^®^ LDEV-Free Reduced Growth Factor Basement Membrane Matrix, Invitrogen, Waltham, MA, USA) on a polyHEMA-coated 96-well plate. The plate was incubated at 37 °C for 30 min, and then 120 µL of complete medium was added to the cells. After 10 days, cells were stained with 2 µg/mL calcein (Life Technologies, Carlsbad, CA, USA) for 30 min at 37 °C. Cell morphology was observed using a confocal laser-scanning microscope (TSP2 Leica, Wetzlar, Germany) interfaced with a Leica DMIRE2 fluorescent.

For chemotaxis experiments, cells were starved for 3 h at 37 °C in serum-free DMEM media; 100,000 cells were placed on the upper layer of a cell culture insert with porous membrane (6.5 mm Corning^®^ Transwell^®^ Inserts, Corning, NY, USA). Before adding cells, the membrane was coated on its bottom layer for 1 h at 37 °C with fibronectin (5 µg/mL), which facilitates cell adhesion and migration. Following 4 h and 30 min of incubation at 37 °C, the porous membrane was fixed in 4% PFA and stained in Crystal Violet. Images of cells that migrated through the membrane were acquired using a stereo microscope. Images were analysed using ImageJ. Details regarding the number of technical and biological replicates are listed in the figure legends.

### 4.8. Statistical Analysis

We performed statistical analysis using GraphPad PRISM software (Version 9.1.1, GraphPad, Inc., San Diego, CA, USA) using two or three-way ANOVA when comparing two or three groups, respectively. Difference was considered significant at *p* < 0.05 (* *p* ≤ 0.05, ** *p* ≤ 0.01). Details regarding the number of technical and biological replicates are listed in the figure legends.

## 5. Conclusions

To our knowledge, our current work represents the first study in which *LINC01605* potential oncogenic function is explored in cancer cells and in which we demonstrate that *LINC01605* participates in the mut_p53 pro-tumorigenic phenotype by regulating cancer cell adhesion and migration. Further characterisation of this regulatory axis may lead to the identification of potential targets, including *LINC01605*, to overcome the mut_p53 oncogenic role in different cancer types.

## Figures and Tables

**Table 1:** Results of Gene Set Enrichment Analysis (GSEA). The table shows the pathways differentially enriched in *mut_TP53*-silenced or *LINC01605*-KO cells. In bold, the pathways that are in common between *mut_TP53*-silenced and in *LINC01605*-KO experiments.

	*mutTP53-silenced*	*LINC01605*-KO
**HALLMARK_OXIDATIVE_PHOSPHOYLATION**	enriched	enriched
**HALLMARK_ADIPOGENESIS**	enriched	enriched
**HALLMARK_CHOLESTEROL_HOMEOSTASIS**	enriched	enriched
**HALLMARK_DNA_REPAIR**	enriched	enriched
**HALLMARK_XENOBIOTIC_METABOLISM**	enriched	enriched
**HALLMARK_EPITHELIAL_MESENCHYMAL_TRANSITION**	enriched	enriched
**HALLMARK_PROTEIN_SECRETION**	enriched	enriched
**HALLMARK_UV_RESPONSE_UP**	enriched	enriched
**HALLMARK_FATTY_ACID_METABOLISM**	enriched	enriched
**HALLMARK_PEROXISOME**	enriched	enriched
**HALLMARK_UNFOLDED_PROTEIN_RESPONSE**	enriched	enriched
**HALLMARK_MYOGENESIS**	enriched	enriched
**HALLMARK_MTORC1_SIGNALING**	enriched	enriched
HALLMARK_HYPOXIA	enriched	
HALLMARK_GLYCOLYSIS	enriched	
HALLMARK_P53_PATHWAY	enriched	
HALLMARK_APOPTOSIS	enriched	
HALLMARK_ESTROGEN_RESPONSE_LATE	enriched	
HALLMARK_ESTROGEN_RESPONSE_EARLY	enriched	
HALLMARK_TNFA_SIGNALING_VIA_NFKB	enriched	
HALLMARK_E2F_TARGETS	enriched	
HALLMARK_HEME_METABOLISM	enriched	
HALLMARK_IL2_STAT5_SIGNALING	enriched	
HALLMARK_COAGULATION	enriched	
HALLMARK_COMPLEMENT	enriched	
HALLMARK_ANDROGEN_RESPONSE	enriched	
HALLMARK_REACTIVE_OXYGEN_SPECIES_PATHWAY	enriched	
HALLMARK_UV_RESPONSE_DN	enriched	
HALLMARK_PI3K_AKT_MTOR_SIGNALING	enriched	
HALLMARK_TGF_BETA_SIGNALING	enriched	
HALLMARK_BILE_ACID_METABOLISM	enriched	
HALLMARK_ANGIOGENESIS	enriched	
HALLMARK_PANCREAS_BETA_CELLS	enriched	
HALLMARK_MYC_TARGETS_V1	enriched	
HALLMARK_MYC_TARGETS_V2	enriched	
HALLMARK_APICAL_JUNCTION		enriched
HALLMARK_IL6_JAK_STAT3_SIGNALING		enriched

## Data Availability

The datasets generated during and analysed during the current study are available in the Sequence Read Archive (SRA) of the National Library of Medicine https://www.ncbi.nlm.nih.gov/sra (accession code: PRJNA815968). The list of samples and web links of RNA-seq samples uploaded in the Sequence Read Archive are shown in Supplementary Table S2. All data generated or analysed during this study are included in this published article (and its Supplementary Information files).

## Supplementary Materials

The supporting information can be downloaded at https://www.mdpi.com/article/10.3390/ijms241813736/s1.
