# Supplementary material for: LINC01605 Is a Novel Target of Mutant p53 in Breast and Ovarian Cancer Cell Lines
Source: Int J Mol Sci. 2023 Sep 6;24(18):13736. doi: 10.3390/ijms241813736 (PMC10531163; doi:10.3390/ijms241813736)
Supplement: Supplementary file 1 [file ijms-24-13736-s001.zip › ijms-2577011-supplementary.pdf]

LINC01605 is a novel target of mutant p53 in breast and ovarian cancer cell lines

Michela Coan*1, Martina Toso*1, Laura Cesaratto1, Ilenia Rigo1, Silvia Borgna1, Anna dalla Pietà1, Luigi Zandonà1, Lorenzo Iuri2, Antonella Zucchetto3, Carla Piazza2, Gustavo Baldassarre1, Riccardo Spizzo1, Milena Sabrina Nicoloso1

1 Division of Molecular Oncology, Department of Translational Research, Centro di Riferimento Oncologico di Aviano (CRO) IRCCS, Via Franco Gallini, 2 33081 Aviano PN, Italy
2 Department of Mathematics, Informatics and Physics, University of Udine, via delle Scienze, 206 33100 Udine, Italy
3 Division of Clinical and Experimental Onco-Hematology, Department of Translational Research, Centro di Riferimento Oncologico di Aviano (CRO) IRCCS, Via Franco Gallini, 2 33081 Aviano PN, Italy

*These authors contributed equally to this work
Corresponding author
Riccardo Spizzo rspizzo@cro.it

Supplementary Figures

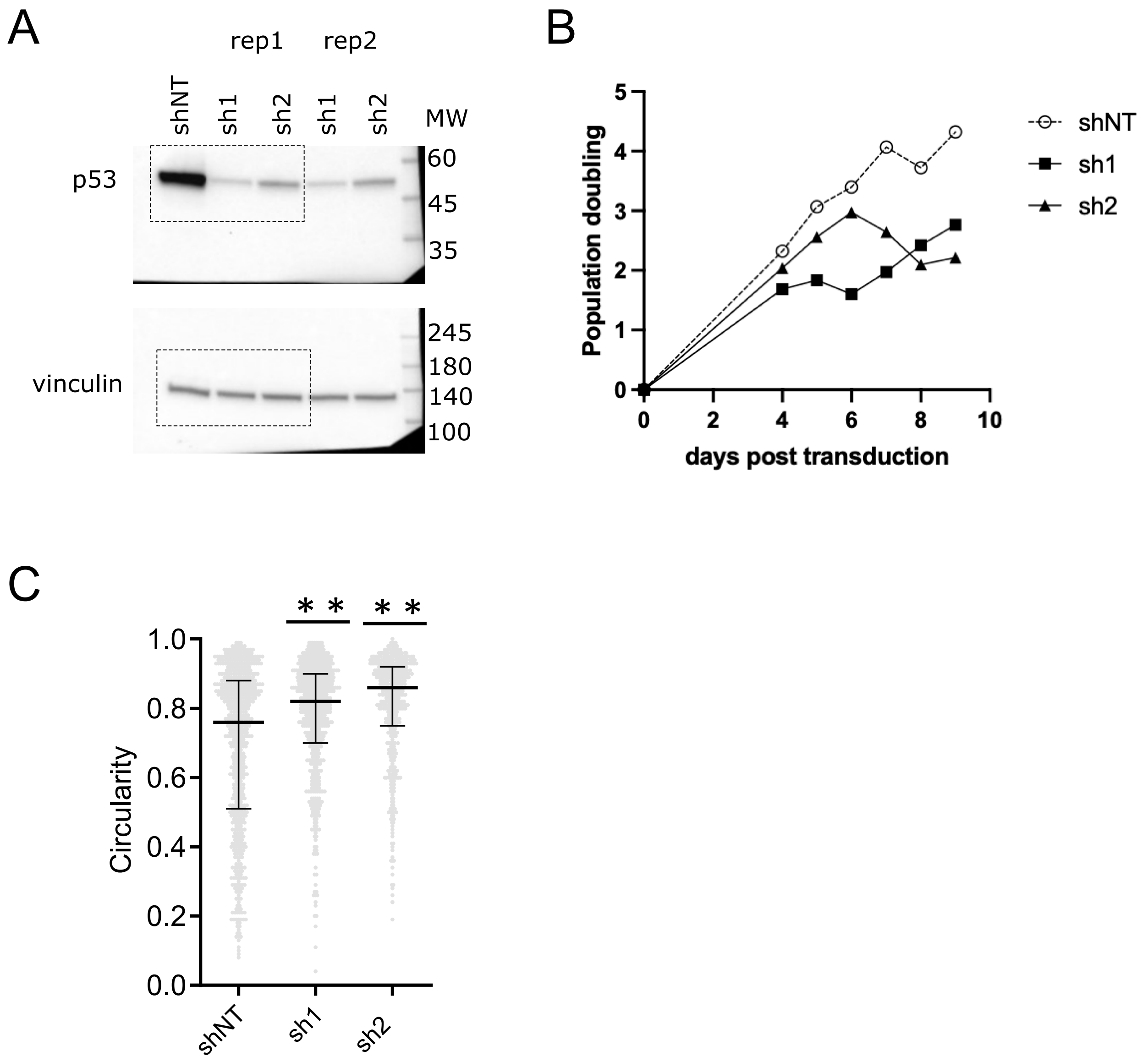

Supplementary Figure S1: Silencing of p53 and circularity of MDA-MB-231 3D colonies. (A) Full-length blots of Figure 1A. Dot lines delineate cropped parts that are shown in Figure 1A. Exposure time was 1 minute. (B) Population doubling experiment. MDA-MB-231 cell lines were plated in 10cm dish after viral transduction. Same number of cells were seeded and counted at different time points. (**C**) Quantification of 3D colony circularity of Figure 1B using ImageJ. ImageJ assigns a value of 1 to circular colonies, whilst values closer to 0 represent less circular and more spread colonies. A total of 10 fields were analysed for each condition. Results are the mean of two biological replicates. (** p-value≤0.01).

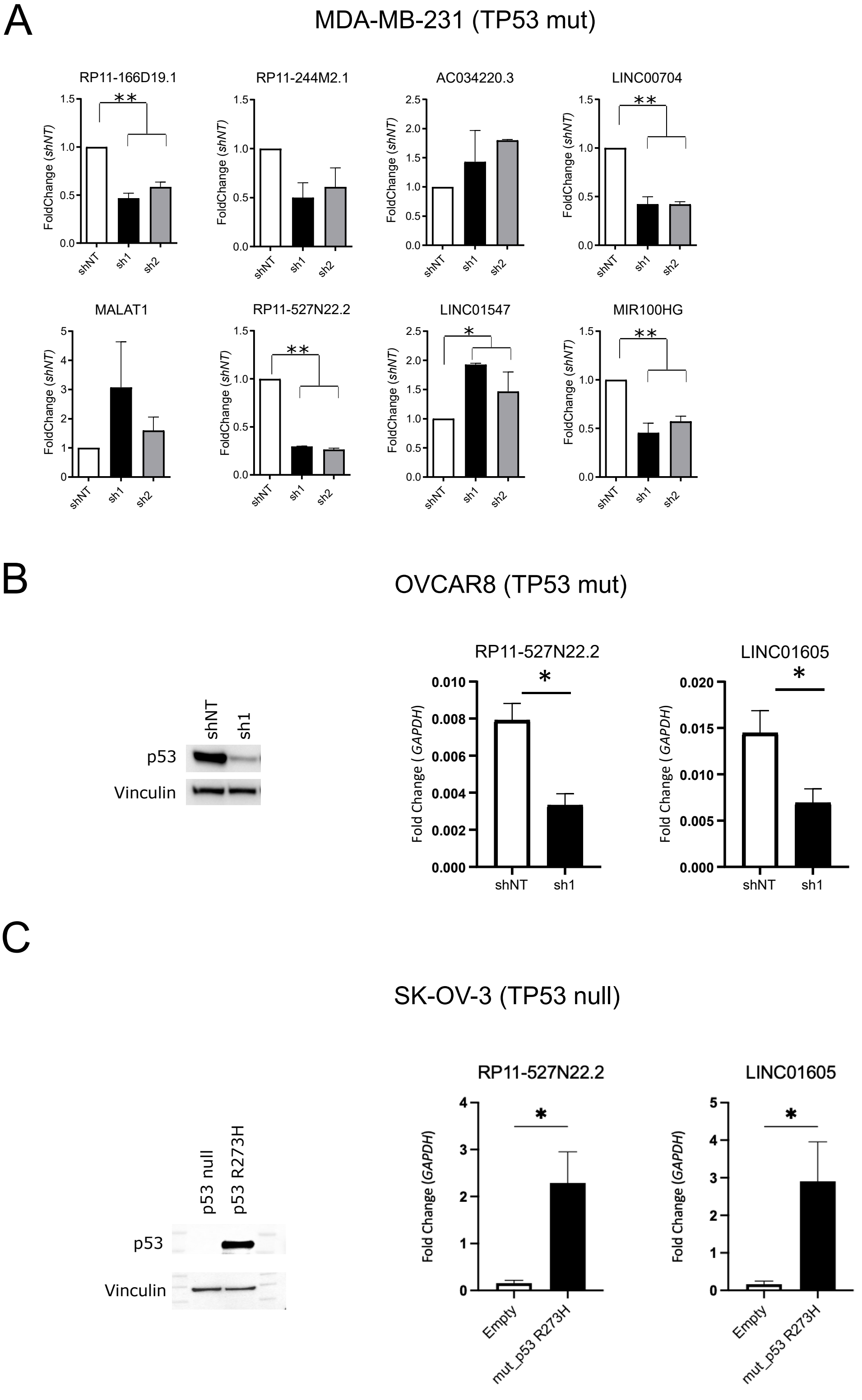

Supplementary Figure S2: Gene expression of candidate lncRNAs in MDA-MB-231, OVCAR8 and in SKOV3 cell lines. **(A)** qRT-PCR of candidate lncRNAs in shNT, sh1 and sh2 MDA-MB-231. **(B)** Mut_p53 protein levels (left) and *LINC01605* and *RP11-57N22.2* expression levels by qRT-PCR (right) in OVCAR8 upon mut_*TP53* silencing. **(C)** Mut_p53 protein levels (left) and *LINC01605* and *RP11-57N22.2* expression levels by qRT-PCR (right) in SKOV3 upon overexpression of mut_*TP53*. (* p-value ≤0.05, ** p-value≤0.01).

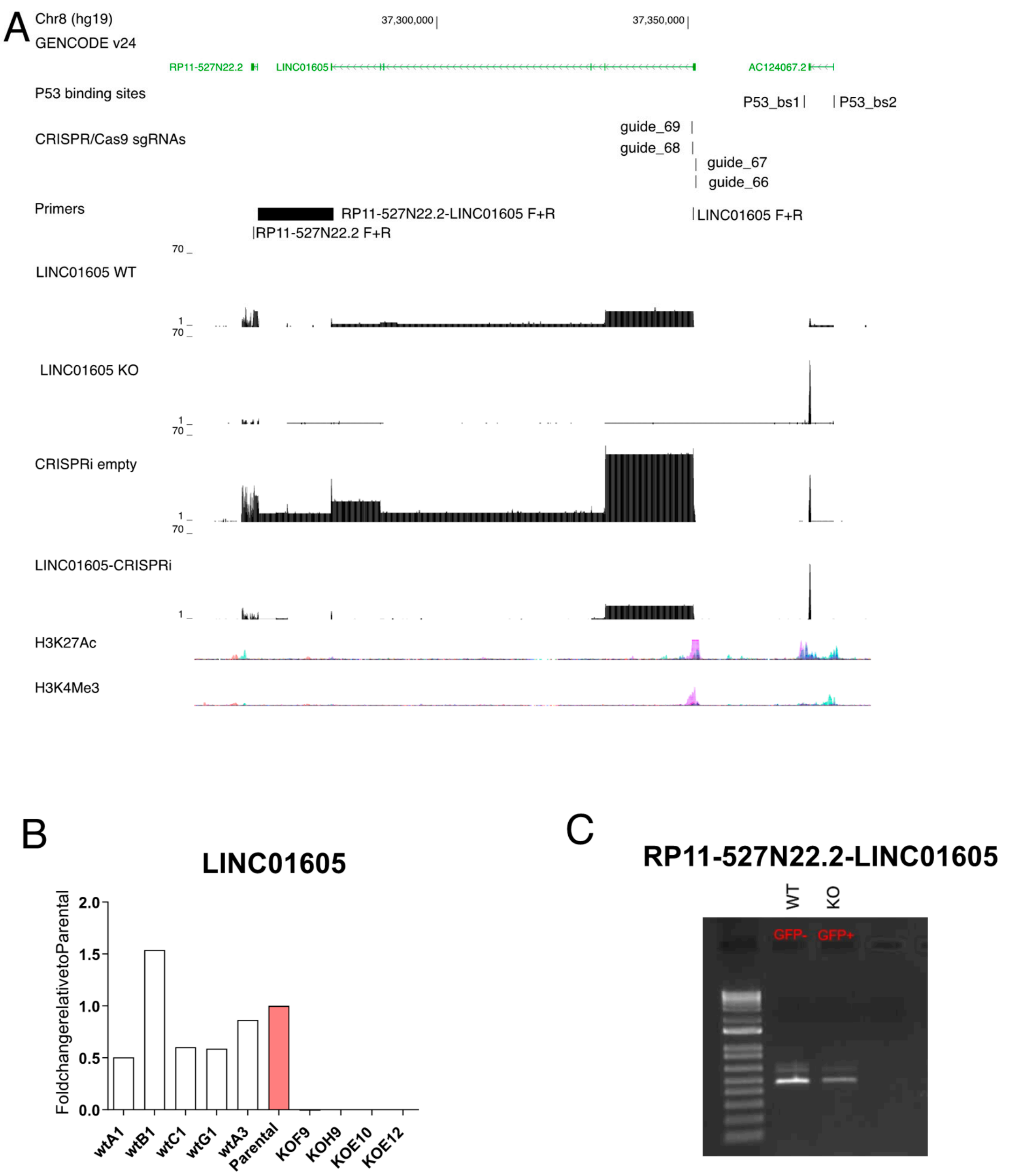

Supplementary Figure S3: Deleting the first exon of LINC01605 in MDA MB 231 cell lines. (**A**) UCSC Genome browser session showing from top to bottom: candidate binding sites of *mut_p53* (p53_bs1 and p53_bs2); single guideRNAs used to knock-out the first exon and epigenetically inhibit the promoter of *LINC01605*; genomic locations of primers used for *LINC01605* and *RP11-527N22.2* RT-qPCR and qPCR screening; RNA-seq sequencing reads for: *LINC01605* WT and KO MDA-MB-231 single clones, CRISPRi empty and CRISPRi-*LINC01605* cell pools. (**B**) qPCR screening for *LINC01605* on genomic DNA obtained from different single MDA-MB-231 clones WT and KO for *LINC01605*. **(C)** Agarose gel electrophoresis showing PCR products of RP11-527N22.2-LINC01605 primers in cDNA of MDA-MB-231 cells transduced with CRISPR-*LINC01605* KO and sorted for GFP- cells (i.e. WT) or GFP+ (i.e. *LINC01605* KO enriched).

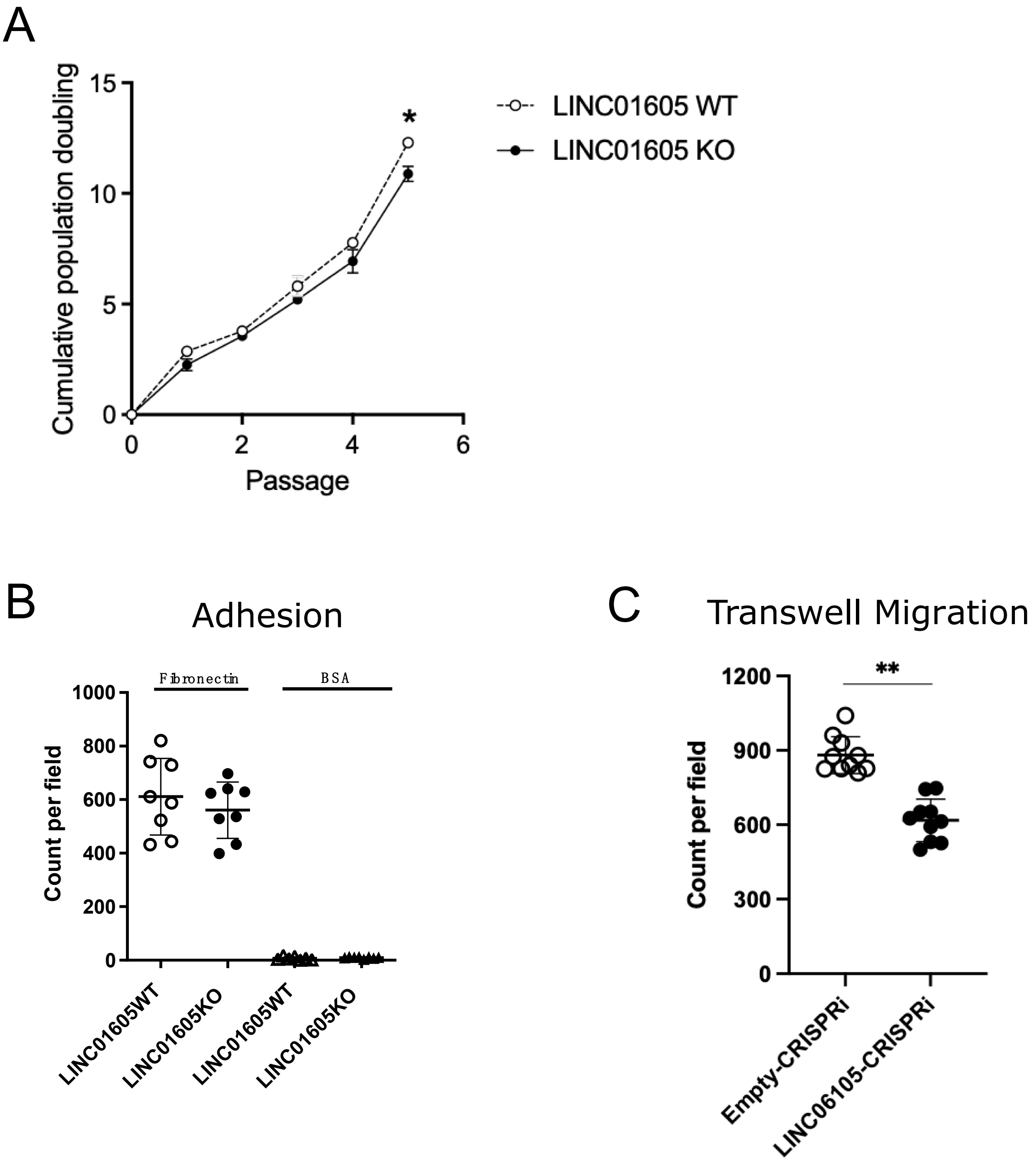

Supplementary Figure S4: *LINC01605* oncogenic role. (A) Population doubling experiments for *LINC01605*-KO and *LINC01605*-WT cells. **(B)** Adhesion assays on fibronectin-coated plates comparing WT and *LINC01605*-KO cells. **(C)** Transwell migration of *LINCO1605*-CRISPRi cells. Count of migrated cells through Boyden chamber in MDA-MB-231 *LINC01605*-CRISPRi cells. Images were acquired with a stereomicroscope. Fifteen different fields were acquired for each cell line and for each biological replicate (three independent biological replicates). (* p-value ≤0.05, ** p-value≤0.01).

Supplementary Table S1. List of cell lines, vectors and primers

Cell type	Source	Species/tumor type	Media
293FT	Thermo Fisher Scientific	H. sapiens	DMEM, 10%FBS, Gln, NaP, NEAA
MDA-MB-231	ATCC	H. sapiens/Breast Cancer	RPMI, 10% FBS, NaP
OVCAR8	ATCC	H. sapiens/Ovarian Cancer	RPMI, 10% FBS, 0.023U/ml Insulin
SKOV-3	ATCC	H. sapiens/Ovarian Cancer	RPMI, 10% FBS, NaP
**Vector**	**Application**	**Source**	
pLV hUbC-Cas9-T2A-GFP	LINC01605-KO	Addgene (#53190)	
pLV hU6-sgRNA hUbC-dCas9-KRAB-T2a-GFP	LINC01605-CRISPRi	Addgene (#71237)	
pLKO shTP53-NT	p53-silencing (not-targeting control)	Sigma (SHCO16)	
pLKO shTP53-1	p53-silencing	Sigma (TRCN0000003756)	
pLKO shTP53-2	p53-silencing	Sigma (TRCN0000003753)	
pCMV-Neo-Bam p53 R273H	mut_p53 overexpression	[38]	
**Guide RNA Name**	**Guide RNA Sequence**		
guide_66	GGGCCGAAATGTATTGGCTG		
guide_67	ACGGGGTTTAGTCACATCTG		
guide_68	TCTGAGTTACACACGCGGAG		
guide_69	GCACCGTCTTGCCCCATGCG		
**Primer Name**	**Primer Sequence**		
Miseq_LINC01605_F	ACCTCCGGAATCACAGTACG		
Miseq_LINC01605_R	CTCCTAGTTCTGCCCTGACG		
P53_bs1 F	AGAAGGGAGTGCTTCCTTAGTG		
P53_bs1 R	AAATGCTTCCAGAAGGAATGAA		
P53_bs2 F	CCAGACTTTGGGGTCCAG		
P53_bs2 R	CTGGGGCCCATCAATCAC		
LINC01605_F	TGAAGGAAGAGAAGGCTGGA		
LINC01605_R	GAGGGAGGAAGCCTGAAAGT		
RP11-527N22.2-LINC01605 F	CATGTGCCAAACGCTGTTC		
RP11-527N22.2-LINC01605 R	ATGGCTGTCCCACAGGC		
RP11-527N22.2_F	ACAAGGCGAGGACAAGAAGA		
RP11-527N22.2_R	CAGATTCTGCCCTTCGCTAC		
PRE1.1 F	CCTGCCACAGAAATCTGCAT		
PRE1.1 R	GTTCACAGTGTGGGTGATGG		
PRE1.2 F	CTGGGGAGTCTCTGCATCTC		
PRE1.2 R	GCTCTGCAACTTTGTGGTTG		
PRE1.3 F	GTGGTGCCTCTGCTCTTCTC		
PRE1.3 R	CACCAGCTATCCAGCACAGA		

PRE1.4 F	GACCATGGCTGAGGCTAAAG
PRE1.4 R	ACATCCTGGTGAAGCAAAGG
PRE1.5 F	TTCCATATGCCGTTGTGTGT
PRE1.5 R	CGGCAGTGTTTGTATTGGAG
PRE1.6 F	CCGGATGGACAGAGACAGAT
PRE1.6 R	TGCCAGTTATGGTGCAAAAG
PRE2 F	CTGCCAGCGTACCTTGTGTA
PRE2 R	AACCTCCCAAGAGCCAATTT
PRE_1A_cloning_SalI_F	acgcgtcgacCAGCCATGGTCACCAGCTAT
PRE_1A_cloning_SalI_R	acgcgtcgacAGTGGGAAGGTTTTGACAGAGA
PRE_1B_cloning_SalI_F	acgcgtcgacACCAGGGCTTTCCCCAAT
PRE_1B_cloning_SalI_R	acgcgtcgacCTTGCACAGGAAAGGCTATTT
PRE_1C_cloning_SalI_F	acgcgtcgacAAGGAAGTGAGGTGACATGC
PRE_1C_cloning_SalI_R	acgcgtcgacCCAGCCTCTTCCATGACATC
PRE_1D_cloning_SalI_F	acgcgtcgacCAGACACACATGCACATATAGACA
PRE_1D_cloning_SalI_R	acgcgtcgacTTGAATGGCAAGGGCTTTTA
PRE_2A_cloning_SalI_F	acgcgtcgacACACAGCTTAAGAGGTAGGAGGA
PRE_2A_cloning_SalI_R	acgcgtcgacCGATGTCAAACTAAGATAATTATGGA
PRE_2B_cloning_SalI_F	acgcgtcgacAAAATTAAGCGGAAGCTCCA
PRE_2B_cloning_SalI_R	acgcgtcgacTGGAATCTGGACTTTCCATTC
LINC01605_Ex1_cloning_KpnI_F	cggggtaccTAGAATGGTGTCTGGGATTCTG
LINC01605_Ex1_cloning_KpnI_R	cggggtaccCCACATGACACTGAAATGCT

Supplementary Table S2. List of samples and web links of RNA-seq samples uploaded in the Sequence Read Archive.

Database	Run	Cell line	Biosample	Replicate/Clone	Link
Sequence Read Archive	SRR18355704	MDA-MB-231	shNT	rep 1	https://trace.ncbi.nlm.nih.gov/Traces/index.html?view=run_browser&page_size=10&acc=SRR18355704&display=metadata
Sequence Read Archive	SRR18355706	MDA-MB-231	shNT	rep 2	https://trace.ncbi.nlm.nih.gov/Traces/index.html?view=run_browser&acc=SRR18355706&display=metadata
Sequence Read Archive	SRR18355701	MDA-MB-231	sh-TP53 1	rep 1	https://trace.ncbi.nlm.nih.gov/Traces/index.html?view=run_browser&acc=SRR18355701&display=metadata
Sequence Read Archive	SRR18355707	MDA-MB-231	sh-TP53 1	rep 2	https://trace.ncbi.nlm.nih.gov/Traces/index.html?view=run_browser&acc=SRR18355707&display=metadata
Sequence Read Archive	SRR18355705	MDA-MB-231	sh-TP53 2	rep 1	https://trace.ncbi.nlm.nih.gov/Traces/index.html?view=run_browser&acc=SRR18355705&display=metadata
Sequence Read Archive	SRR18355708	MDA-MB-231	sh-TP53 2	rep 2	https://trace.ncbi.nlm.nih.gov/Traces/index.html?view=run_browser&acc=SRR18355708&display=metadata
Sequence Read Archive	SRR18355699	MDA-MB-231	WT	clone 1	https://trace.ncbi.nlm.nih.gov/Traces/index.html?view=run_browser&acc=SRR18355699&display=metadata
Sequence Read Archive	SRR18355700	MDA-MB-231	WT	clone 2	https://trace.ncbi.nlm.nih.gov/Traces/index.html?view=run_browser&acc=SRR18355700&display=metadata
Sequence Read Archive	SRR18355709	MDA-MB-231	WT	clone 3	https://trace.ncbi.nlm.nih.gov/Traces/index.html?view=run_browser&acc=SRR18355709&display=metadata
Sequence	SRR183	MDA-	WT	clone 4	https://trace.ncbi.nlm.nih.gov/Traces/index.html?view=run_browser&a

Read Archive	55710	MB-231			cc=SRR18355710&display=metadata
Sequence Read Archive	SRR18355702	MDA-MB-231	KO LINC01605	clone 1	https://trace.ncbi.nlm.nih.gov/Traces/index.html?view=run_browser&acc=SRR18355702&display=metadata
Sequence Read Archive	SRR18355703	MDA-MB-231	KO LINC01605	clone 2	https://trace.ncbi.nlm.nih.gov/Traces/index.html?view=run_browser&acc=SRR18355703&display=metadata
Sequence Read Archive	SRR18355697	MDA-MB-231	KO LINC01605	clone 3	https://trace.ncbi.nlm.nih.gov/Traces/index.html?view=run_browser&acc=SRR18355697&display=metadata
Sequence Read Archive	SRR18355698	MDA-MB-231	KO LINC01605	clone 4	https://trace.ncbi.nlm.nih.gov/Traces/index.html?view=run_browser&acc=SRR18355698&display=metadata
